# TNF-α promotes extracellular vesicle release in mouse astrocytes through glutaminase

**DOI:** 10.1186/s12974-017-0853-2

**Published:** 2017-04-20

**Authors:** Kaizhe Wang, Ling Ye, Hongfang Lu, Huili Chen, Yanyan Zhang, Yunlong Huang, Jialin C. Zheng

**Affiliations:** 1 Center for Translational Neurodegeneration and Regenerative Therapy, Shanghai Tenth People’s Hospital affiliated to Tongji University School of Medicine, Shanghai, 200072 China; 2 Department of Immunology, Tongji University School of Medicine, Shanghai, 200092 China; 3 Collaborative Innovation Center for Brain Science, Tongji University, Shanghai, 200092 China; 4 Departments of Pharmacology and Experimental Neuroscience, University of Nebraska Medical Center, Omaha, NE 68198-5930 USA; 5 Department of Pathology and Microbiology, University of Nebraska Medical Center, Omaha, NE USA

**Keywords:** TNF-α, Glutaminase, Extracellular vesicles, Astrocytes

## Abstract

**Background:**

Extracellular vesicles (EVs) are membrane-contained vesicles shed from cells. EVs contain proteins, lipids, and nucleotides, all of which play important roles in intercellular communication. The release of EVs is known to increase during neuroinflammation. Glutaminase, a mitochondrial enzyme that converts glutamine to glutamate, has been implicated in the biogenesis of EVs. We have previously demonstrated that TNF-α promotes glutaminase expression in neurons. However, the expression and the functionality of glutaminase in astrocytes during neuroinflammation remain unknown. We posit that TNF-α can promote the release of EVs in astrocytes through upregulation of glutaminase expression.

**Results:**

Release of EVs, which was demonstrated by electron microscopy, nanoparticle tracking analysis (NTA), and Western Blot, increased in mouse astrocytes when treated with TNF-α. Furthermore, TNF-α treatment significantly upregulated protein levels of glutaminase and increased the production of glutamate, suggesting that glutaminase activity is increased after TNF-α treatment. Interestingly, pretreatment with a glutaminase inhibitor blocked TNF-α-mediated generation of reactive oxygen species in astrocytes, which indicates that glutaminase activity contributes to stress in astrocytes during neuroinflammation. TNF-α-mediated increased release of EVs can be blocked by either the glutaminase inhibitor, antioxidant *N*-acetyl-l-cysteine, or genetic knockout of glutaminase, suggesting that glutaminase plays an important role in astrocyte EV release during neuroinflammation.

**Conclusions:**

These findings suggest that glutaminase is an important metabolic factor controlling EV release from astrocytes during neuroinflammation.

**Electronic supplementary material:**

The online version of this article (doi:10.1186/s12974-017-0853-2) contains supplementary material, which is available to authorized users.

## Background

In the central nervous system (CNS), abnormal protein aggregation, autoimmunity, and infection can lead to neurodegenerative conditions, such as amyotrophic lateral sclerosis (ALS) [1], Alzheimer’s disease (AD) [2], Parkinson’s disease (PD) [3], and primary progressive multiple sclerosis (PPMS) [4]. Inflammation within the CNS, where reactive glia shift toward a proinflammatory phenotype, is common to these neurodegenerative diseases. As the resident immune cells in the CNS, glial cells release cytokines, chemokines, as well as potentially neurotoxic substances including excess levels of glutamate, nitric oxide, and arachidonic acid [5] during disease states. Cytokines, especially TNF-α, are typically elevated during neurodegenerative disease states and further promote CNS inflammation [6]. TNF-α is known to exert both homeostatic and pathophysiological roles in the central nervous system by regulating immunologic and metabolic states of astrocytes, the main glial cell type in the CNS [7, 8].

Glutamate metabolism is one of the major metabolic pathways in the CNS. Glutaminase (GA; EC 3.5.1.2), an enzyme localized in the inner membrane of the mitochondria, is a rate-limiting enzyme in glutamate metabolism that catalyzes the conversion of glutamine to glutamate and ammonia [9]. Glutaminase is abundantly expressed in the brain. The main cell types for glutaminase expression include neurons, but microglia, macrophages, and astrocytes are also known to express glutaminase [10]. The mammalian GA family members are encoded by two paralogous genes, *Gls* and *Gls2*, presumably derived from a common ancestral gene by duplication and divergent evolution [11]. Furthermore, two glutaminase (GLS) allozymes, glutaminase C (GAC) and kidney type glutaminase (KGA), are from two different transcripts and both are expressed in the brain. Research on GLS has long been focused on neurotoxicity and cancer. Recent evidence indicates that GLS might also be important in intercellular communication through extracellular vesicles [12–14].

Extracellular vesicles (EVs) are membrane-contained vesicles that include exosomes, microvesicles, and apoptotic bodies [15]. EVs can be released into interstitial fluid, cerebrospinal fluid (CSF) [16], circulating blood [17], urine [18], lymph [19], and glandular secretions, asserting functions to nearby or distant cells [20]. Almost all body cells release EVs that fuse with target cells, by which proteins, lipids, or nucleic acids are transferred from cell to cell. Therefore, EVs are important mediators of cell-to-cell communication [21]. EVs are abundant in the CNS and are thought to facilitate the intercellular communication, maintenance of myelination, synaptic plasticity, antigen presentation, and tropic support of neurons [22, 23]. Therefore, it is of great significance to study the mechanism of EV release in order to understand the development and progression of CNS inflammation.

To determine the mechanism of EV release during neuroinflammation, we used TNF-α to stimulate primary mouse astrocytes. TNF-α induced the increased release of EVs and elevated protein levels of GLS in mouse astrocytes. Furthermore, TNF-α induced the generation of reactive oxygen species (ROS) in astrocytes through GLS. Treatment with either a glutaminase inhibitor, an antioxidant *N*-acetyl-l-cysteine, or using astrocytes that had genetic knockout of glutaminase (Gls−/−) reduced TNF-α-mediated EV release, suggesting that glutaminase is required for EV release in astrocytes during neuroinflammation. Understanding the mechanism of EV release in astrocytes during neuroinflammation is important in identifying novel therapeutic targets in the relevant neurological diseases.

## Methods

### Animals and reagents

C57BL/6J mice were housed and maintained in the Comparative Medicine Facility of the Tongji University School of Medicine (Shanghai, China). All procedures were conducted in accordance with the protocols approved by the Institutional Animal Care and Use Committee at the Tongji University School of Medicine. Postnatal (P1 to P2) brain tissues were used for mouse astrocyte cultures. Gls knock in mouse embryonic stem (ES) cells were obtained from the Knockout Mouse Project (KOMP) Repository (CA, USA). Recombinant mouse TNF-α and IL-1β were obtained from R&D Systems. *N*-Acetyl-l-cysteine (NAC, A7250), 6-Diazo-5-oxo-l-norleucine (L-DON, D2141), BPTES (SML0601), and GW4869 (D1692) were obtained from Sigma-Aldrich.

### Isolation and culture of primary mouse astrocytes

Cortices of C57BL/6J mice were dissected and mechanically dissociated using forceps to remove the membranes and large blood vessels. Brain tissues were digested by Trypsin-EDTA (Life Technologies) and then plated on cell culture flasks in Dulbecco’s modified Eagle medium Nutrient Mixture F-12 (DMEM/F-12). Culture medium was supplemented with FBS (10% *v*/*v*) and penicillin/streptomycin (1% *v*/*v*). Cultures were maintained in a humidified chamber (37 °C, 5% CO_2_ incubator). After 7 to 10 days, the astrocytes were harvested by trypsinization.

### Isolation of EVs

The method for the isolation of extracellular vesicles has been described previously [24]. EVs were isolated from the serum-free culture of mouse astrocytes through differential centrifugation. Briefly, the supernatants were first centrifuged at 300 × *g* for 10 min to remove free cells, at 3000 × *g* for 20 min to remove cellular debris, and then 10,000 × *g* for 30 min to remove intracellular organelles. Lastly, EVs were collected by ultracentrifugation at 100,000 × *g* for 2 h at 4 °C. To prepare EVs for western blot, the EVs pellets were lysed in M-PER mammalian protein extraction reagent (Thermo Scientific, Pittsburgh, PA).

### Dynamic light scattering

Extracellular vesicles were characterized at 25 °C using Nano ZS90 (Malvern Instruments, UK). Eighty microliter samples were loaded into a microcuvette (ZEN0118, Malvern Instruments, UK) for measurement.

### Nanoparticle tracking analysis (NTA)

The size and number of extracellular vesicles were assessed with NanoSight NS300 system (Malvern Instruments, UK). Astrocytes were cultured in 6-cm culture dishes. At 24 h after medium change, EVs were isolated from normalized volumes of serum-free culture supernatants through differential centrifugation and resuspended with 150 μl PBS. The supernatant was diluted at 1:100 in PBS, and 1 ml solution was used for NanoSight analysis.

### Scanning electron microscopy (SEM)

Mouse astrocytes were grown on a glass coverslip, fixed with 2.5% glutaraldehyde, and washed three times with PBS. The cells were then dehydrated in a series of increasing ethanol concentrations and transferred for critical drying. After coating with platinum/palladium using a sputter coater, the sample was imaged with a scanning electron microscope (S-3400, Hitachi).

### Transmission electron microscopy (TEM)

EVs were negatively stained and then spread on the copper grids. The droplets of EVs were removed with filter paper and air-dried at room temperature. Images were obtained using transmission electron microscopy (JEM-1230, JEOL Ltd.).

### Western Blot

Cells or EV pellets were lysed in M-PER mammalian protein extraction reagent (Thermo Scientific). Proteins from lysates were separated by sodium dodecyl sulfate-polyacrylamide gel electrophoresis (SDS-PAGE). After electrophoretic transfer to polyvinyldifluoridene (PVDF) membranes (Millipore, Billerica, MA, USA), proteins were treated with purified primary antibodies for glutaminase (1:1000; Abcam), GFAP (1:1000; Cell Signaling Technologies), β-actin (1:5000; Sigma Aldrich), flotillin-2 (1:5000; BD biosciences), and ALG-2 interacting protein (Alix) (1:1000; Cell Signaling Technologies) overnight at 4 °C followed by a horseradish peroxidase-linked secondary anti-rabbit or anti-mouse antibody (1: 5000; Icllab). Antigen–antibody complexes were visualized by Pierce ECL Western Blotting Substrate. Mitochondrial protein was isolated by the Mitochondria Isolation Kit for Cultured Cells (ab110171, Abcam). ATP5A (1:1000; Abcam) was used as a mitochondrial marker to indicate that there is no mitochondria contamination in the cytosol.

### Immunocytochemistry

Mouse astrocytes cultured on cover glasses were fixed with 4% PFA, rinsed with PBS, and then blocked by 2% BSA in PBS. Cells were incubated overnight at 4 °C with primary antibodies anti-GFAP (1:1000; Abcam). Cover glasses were washed and incubated for 1 h at room temperature with secondary antibodies including anti-mouse IgG (coupled with Alexa Fluor 488, Life Technologies). Nuclear DNA was stained with DAPI. Cover glasses were mounted on glass slides with mounting buffer (Sigma-Aldrich). Morphological changes were visualized by a Zeiss 710 confocal laser scanning microscope.

### ROS measurement

ROS measurement was assayed by dichloro-dihydro-fluorescein diacetate (DCFH-DA). Mouse astrocytes were incubated in 10 μM DCFH-DA (50101, YEASEN) for 30 min at 37 °C, 5% CO_2_ and then were washed with PBS. The ROS were determined using conventional fluorescence microscopy (ZEISS).

### Intracellular and extracellular glutamate analysis

Intracellular glutamate detection was performed with the Amplex Red Glutamic Acid/Glutamate Oxidase Assay Kit from Invitrogen following manufacturer’s procedure. High performance liquid chromatography (HPLC) analysis for extracellular glutamate was performed as previously described [25].

### Cell viability measurement

Briefly, mouse astrocytes were treated with TNF-α for 24 h, the cell viability was assayed by cell viability assay kit (Promega, G7570). The luminescent signal recording from the reaction was as the standard of cell viability.

### Statistical analyses

Data were evaluated statistically by the analysis of variance (ANOVA), followed by Tukey’s test for multiple comparisons. Data were shown as mean ± SD. *, **, and *** denote *p* < 0.05, *p* < 0.01, and *p* < 0.001 in comparison to control, respectively. ^#^, ^##^, and ^###^ denote *p* < 0.05, *p* < 0.01, and *p* < 0.001 in comparison to TNF-α-treated groups, respectively.

## Results

### TNF-α increases EV levels in mouse astrocyte cultures

**Fig. 1:**
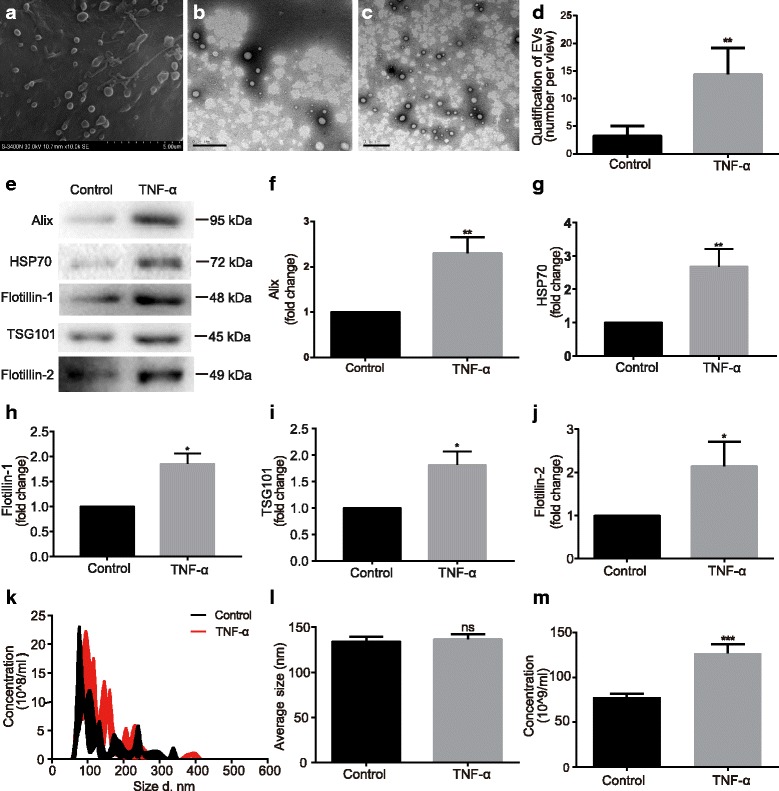
TNF-α stimulation increases EV release from mouse astrocytes. **a** Scanning electron microscopy (SEM) of mouse astrocytes. **b**, **c** EVs were isolated from serum-free cultural supernatants and observed under TEM using negative staining. *Scale bar* 500 nm. Representative TEM images of EVs derived from control and TNF-α-treated astrocytes. **d** EV numbers were quantified by manually counting from a total of nine random vision fields. **e** EVs were isolated from normalized volumes of supernatants from control and TNF-α-treated culture after 24 h. **f**–**j** Alix, HSP70, Flotillin-1, TSG101, and Flotillin-2 as the markers of EVs were determined by Western Blot. Levels of Alix and Flotillin-2 were compared with control. **k**–**m** Nanometer diameter range, average size, and concentration of the EVs were obtained by NTA. Data were represented as mean ± SD from three independent experiments. *, **, and *** denote *p* < 0.05, *p* < 0.01, and *p* < 0.001 in comparison to control, respectively. ns denotes no significance

Mouse astrocytes were established from the cortices of neonatal mice as previously described (Additional file 1: Figure S1a) [26]. To visualize EV generation in astrocytes, we first used scanning electron microscopy (SEM) on the cultures. SEM revealed polarized membranous structures of mouse astrocytes that are likely EVs in the course of releasing from the plasma membrane (Fig. 1a).

Our previous studies have demonstrated HIV-1 infection and immune activation increase release of EVs from macrophages and microglia [24]. To further assess the relationship between proinflammatory cytokines and the release of extracellular vesicles, we used dynamic light scattering (DLS) analysis, which determined the size distribution of small particles in suspension. In particular, the release of EVs from astrocytes during inflammatory cytokine treatment was examined. By analyzing the signaling intensity (Additional file 1: Figure S1d), we found that EV sizes ranged from 50 to 800 nm, and TNF-α (50 ng/ml) treatment had no effect on the size of EVs. Cell viability did not change after TNF-α treatment (Additional file 1: Figure S1b). To determine quantitative changes of EVs, we isolated them from serum-free supernatants with or without TNF-α treatment for 24 h. The EV pellets were collected and resuspended in PBS for negative staining under TEM (Fig. 1b, c). The numbers of EVs per field under TEM were significantly higher in TNF-α treatment group as compared with that in control (Fig. 1d), suggesting that TNF-α treatment leads to increased release of EVs in mouse astrocytes. Calreticulin, a marker of endoplasmic reticulum, is not present in the 100,000 × *g* fraction, suggesting that cell debris is not present in the fraction (Additional file 1: Figure S1c). EVs were also subjected to Western Blot for specific EVs markers, including ALG-2 interacting protein (Alix), HSP70, Flotillin-1, TSG101, and Flotillin-2 (Fig. 1e). Consistent with TEM data, TNF-α significantly increased the expression levels of Alix (Fig. 1f), HSP70 (Fig. 1g), Flotillin-1 (Fig. 1h), TSG101 (Fig. 1i), and Flotillin-2 (Fig. 1j) in EVs from the treated astrocytes as compared to those from the control. In addition, we also analyzed EVs using NTA tracking. NTA tracking allowed a precise analysis of secreted vesicle size and concentration (Fig. 1k). Consistent with the previous data, TNF-α induced a two-fold increase in the concentrations of EVs, as compared to the control group (Fig. 1m), but the average size of the EVs had no change (Fig. 1l). Together, these data suggest that TNF-α increases EV release in primary mouse astrocytes.

### TNF-α increases GLS expression and promotes glutamate generation in mouse astrocytes

**Fig. 2:**
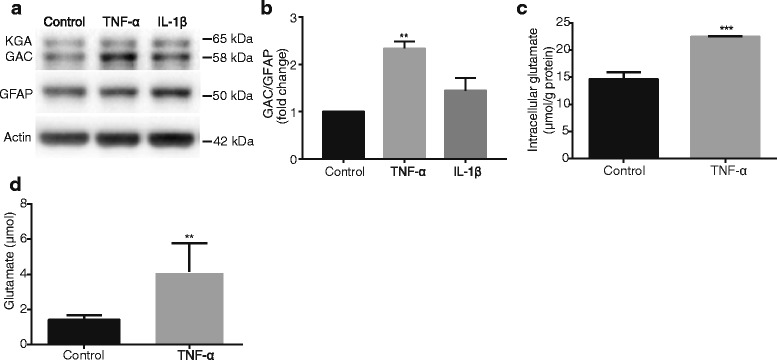
TNF-α regulates GLS protein expression in mouse astrocytes. **a** Mouse astrocytes were treated with IL-1β (10 ng/ml) or TNF-α (50 ng/ml) for 72 h. Whole cell lysates were collected and protein levels of GLS, including two GLS allozymes, kidney type glutaminase (KGA, 65 kDa) and glutaminase C (GAC, 58 kDa), were analyzed by Western Blot. GFAP was a marker of astrocytes. Actin was used as a loading control. **b** Protein levels were normalized as a ratio to GFAP after densitometric quantification and presented as fold change relative to control astrocytes. **c**, **d** Intracellular concentration of glutamate was determined by a Glutamic Acid/Glutamate Oxidase Assay Kit, and the extracellular concentration of glutamate in cell-free supernatants was determined by HPLC. Results were expressed as the mean ± SD from three independent experiments. ** and *** denote *p* < 0.01 and *p* < 0.001 in comparison to control, respectively

Previous studies showed that TNF-α regulates the expression of GLS in human neuron and microglia [5, 27]. Therefore, we further investigated whether glutaminase, particularly its two main isoforms KGA and GAC, is upregulated by TNF-α treatment in mouse astrocytes. Protein levels of GLS isoforms in astrocyte cultures were determined following proinflammatory cytokine treatment (TNF-α 50 ng/ml, IL-1β 10 ng/ml). When protein levels of GLS isoforms were determined by Western Blot, TNF-α induced a 1.8-fold increase of GAC (58 kDa) when compared with the untreated control (Fig. 2a, b), whereas IL-1 β had no significant effect on protein levels of GAC (Fig. 2a, b). Therefore, we used TNF-α to treat mouse astrocytes in the subsequent experiments. To determine whether changes of GLS lead to changes of enzyme activity, we checked the intracellular and extracellular glutamate levels in mouse astrocytes upon TNF-α treatment. As expected, TNF-α dramatically upregulated intracellular glutamate as compared to the untreated control group (Fig. 2c). Similar to the intracellular glutamate level, the extracellular glutamate level also increased significantly following the treatment with TNF-α as compared to the control (Fig. 2d). Interestingly, we found that the location of GLS in mouse astrocytes had changed after TNF-α treatment and GLS were released from the mitochondria to the cytoplasm (Additional file 2: Figure S2a). Together, these data suggest that TNF-α increases GLS expression that leads to an increase of its enzymatic production of glutamate in mouse astrocyte.

### TNF-α induces ROS generation in mouse astrocytes

**Fig. 3:**
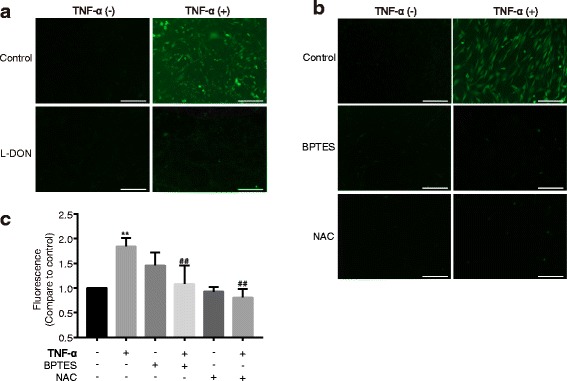
TNF-α induces ROS generation in mouse astrocytes. Mouse astrocytes were pretreated with 1 mM L-DON (**a**) or 10 μM BPTES (**b**, **c**) and 10 mM NAC for 30 min, and then treated with TNF-α (50 ng/ml). After 6 h (L-DON) or 48 h (BPTES, NAC) treatment, cells were stained by loading with 10 μM DCFH-DA for 30 min at 37 °C. Cells were washed with serum-free culture medium, and then subjected to fluorescence microscope (**a**, **b**) and fluorescent microplate reader (**c**). All results were representative of three independent experiments (*n* = 3)

Mitochondria are a major source of intracellular ROS, and are also particularly vulnerable to oxidative stress. Our previous studies suggest that ROS is closely related to mitochondrial permeability [28, 29]. To determine the relationship between oxidative stress and GLS, mouse astrocytes were pretreated with 1 mM L-DON, a glutaminase inhibitor. After 6 h of treatment with or without TNF-α, the ROS level in the cells was detected by 2,7-dichlorodi-hydrofluoresceindiacetate (DCFH-DA), a common indicator for ROS generation in cells [30, 31]. The intracellular ROS was determined by the conversion of non-fluorescence DCFH-DA to a fluorimetric DCF that exhibits peak fluorescence in the green spectrum. TNF-α treatment increased ROS generation in astrocytes as compared with untreated control group (Fig. 3a, b). Furthermore, inhibition of GLS by L-DON, a competitive glutaminase inhibitor, blocked the ROS generation (Fig. 3a). We also used an allosteric GLS inhibitor BPTES, one of the most potent blockers of GLS, to inhibit GLS activity. BPTES suppressed the production of ROS, which increased in TNF-α-treated astrocytes (Fig. 3b, c). In addition, when we pretreated the astrocytes with NAC, a remover of ROS, the ROS levels in TNF-α-treated astrocytes were reversed (Fig. 3b, c). These data suggested that ROS generation is correlated with GLS activity in mouse astrocytes.

### TNF-α induces EV release from mouse astrocytes through GLS

**Fig. 4:**
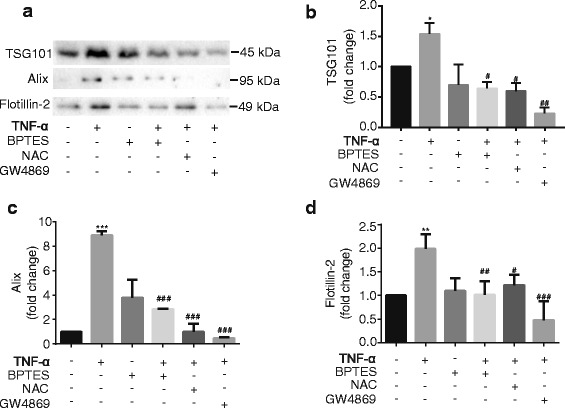
TNF-α induces EV release through GLS in mouse astrocytes. **a** Mouse astrocytes were pretreated with 10 μM BPTES, 10 mM NAC, or 10 μM GW4869 for 1 h, and then treated with TNF-α (50 ng/ml) for 24 h in serum-free media. EVs were isolated from the supernatants, and EV protein lysates were prepared. The levels of TSG101, Alix, and Flotillin-2 were determined by Western Blots. **b**–**d** Densitometric quantifications of the TSG101, Alix, and Flotillin-2 protein levels were presented as fold change relative to the controls. The results shown were representative of three independent experiments. Quantification results were shown as mean ± SD of experiments performed in triplicate (*n* = 3). ** and *** denote *p* < 0.01 and *p* < 0.001 in comparison to control, respectively. ^#^, ^##^, and ^###^ denote *p* < 0.05, *p* < 0.01, and *p* < 0.001 in comparison to TNF-α treated group, respectively

To determine whether GLS participated in the release of EVs in TNF-α-treated mouse astrocytes, we pretreated the cultures with BPTES or GW4869 (a neutral sphingomyelinases inhibitor that halts the biogenesis of EVs for 1 h. After TNF-α treatment, EVs were extracted and then subjected to Western Blot for EV detection (Fig. 4a). After the densitometric quantifications of the EV markers TSG101 (Fig. 4b), Alix (Fig. 4c), and Flotillin-2 (Fig. 4d), we found that they were significantly increased in EVs isolated from TNF-α-treated astrocytes. Both BPTES and GW4869 dramatically inhibited the EV release, and the expression levels of the EV markers decreased significantly when compared with TNF-α group (Fig. 4b–d). Together, these interesting findings indicated that GLS was an important factor participating in TNF-α-mediated EV release.

Previous research has shown that the release of EVs was closely related to ROS [32–34]. In order to further investigate the relationship between ROS and EV release in mouse astrocytes, cells were pretreated with NAC (10 mM) for 1 h, and then EVs were collected from serum-free culture after 24 h treatment. Pretreatment with NAC dramatically reduced the levels of TSG101 (Fig. 4b), Alix (Fig. 4c), and Flotillin-2 (Fig. 4d) in the EV lysates, indicating that the release of EVs in TNF-α group was blocked by NAC (Fig. 4).

Taken together, these data suggested that GLS and the subsequent ROS generation was an important pathway for EV release in mouse astrocytes during TNF-α-mediated inflammation.

### GLS mediates EV release in mouse astrocytes

**Fig. 5:**
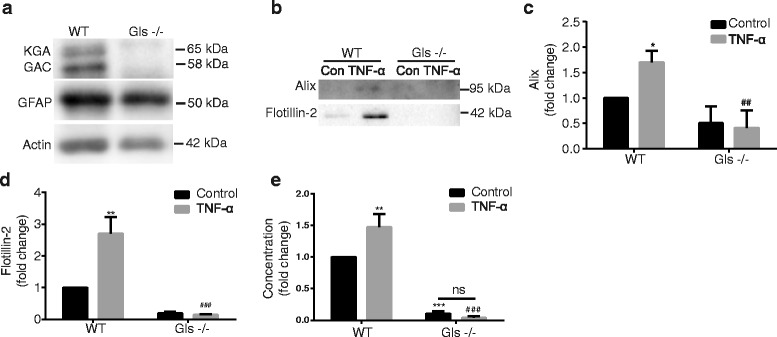
GLS mediates EV release in mouse astrocytes. **a** Wild-type and Gls−/− mouse astrocytes were acquired. Protein levels of GLS, including two GLS allozymes, kidney type glutaminase (KGA, 65 kDa) and glutaminase C (GAC, 58 kDa), were analyzed by Western Blot. GFAP was a marker of astrocytes. Actin was used as a loading control. **b** Wild-type and Gls−/− mouse astrocytes were treated with TNF-α (50 ng/ml) for 24 h in serum-free media. EVs were isolated from the supernatants, and EV protein lysates were prepared. **c**, **d** The levels of Alix and Flotillin-2 were determined by Western Blots. Quantification results of Alix and Flotillin-2 were shown as mean ± SD. EVs were isolated from normalized volumes of serum-free supernatants of control and TNF-α treatment group for 24 h. **e** EVs were isolated and resuspended with PBS, and then the concentration of EVs was analyzed by NTA. *, **, and *** denote *p* < 0.05, *p* < 0.01, and *p* < 0.001 in comparison to control, respectively. ^##^ and ^###^ denote *p* < 0.01 and *p* < 0.001 in comparison to TNF-α treated groups, respectively. ns denotes no significance

To investigate GLS in mediating EV release in mouse astrocytes, Gls knock out (Gls−/−) mice were used. Since Gls−/− mice died shortly after birth [35], Gls−/− astrocytes were obtained at E18.5. Protein samples of Gls−/− astrocytes were used for Western Blot (Fig. 5a) to confirm gene knockout. The ROS production in the GLS knockout mice was detected (Additional file 2: Figure S2b), and the quantification data showed the ROS production had no change with TNF-α stimulation (Additional file 2: Figure S2c). Wild-type and Gls−/− mouse astrocytes were first treated with TNF-α for 24 h, and EVs were extracted for Western Blot (Fig. 5b). After the densitometric quantifications of the Western Blot, the data revealed significantly increased levels of Alix (Fig. 5c) and Flotillin-2 (Fig. 5d) in EV lysates from TNF-α treated wild type cells. Furthermore, after TNF-α treatment, the Gls−/− group had reduced levels of EVs as compared to wild-type cells (Fig. 5c, d). EVs derived from wild-type and Gls−/− mouse astrocytes were also subjected to size-distribution analysis by NTA (Fig. 5e). TNF-α treatment induced a two-fold increase of EVs in the wild type astrocytes. Knockout of GLS induced a ten-fold decrease in EV concentration compared with wild-type mouse astrocytes (Fig. 5e). These findings demonstrated that GLS is important for the release of extracellular vesicles in mouse astrocytes following TNF-α treatment.

Taken together, our data unveiled a relationship between glutaminase and EV release in mouse astrocytes. TNF-α treatment upregulated protein levels of GLS and increased the production of ROS. GLS and ROS could influence each other, and both of them could promote EV release.

## Discussion

In this study, our results present three important new findings regarding EV release. *First*, TNF-α treatment significantly promoted the release of EVs in mouse astrocytes. *Second*, TNF-α upregulated the protein level of glutaminase and pretreatment with a glutaminase inhibitor blocked TNF-α-mediated generation of ROS in astrocytes. *Last*, the TNF-α-mediated increased release of EVs can be inhibited by either the glutaminase inhibitor, antioxidant NAC, or genetic knockout of glutaminase. These observations suggest that GLS may contribute to EV release in mouse astrocytes.

EVs are associated with the message delivery of the nervous system and may involve in the pathogenesis of many neuroinflammatory disorders, both infectious and neurodegenerative [36]. However, the mechanism underlying formation and secretion of EVs is still unclear. EVs are modulated in different cell types by various environmental changes, such as ligand encounter or stress conditions, and could be one of the means used by tissues to adapt to these changes [37]. There have been reports showing that EV release is modulated by induction of ROS [32–34], inflammation and ATP [38], calcium [39], and acid sphingomyelinase [40]. The important observation reported here is that TNF-α increased ROS generation, and that this can be blocked by GLS inhibitors. Glutaminase is usually located in the mitochondria, and our previous study showed that ROS could cause the location change in macrophage [28]. These findings suggest that GLS and ROS can influence each other. More importantly, TNF-α-mediated increased release of EVs can be inhibited by both antioxidant NAC and GLS inhibitors. ROS activates various stress pathways, including proapoptotic p38, p53, and SAPK/JNK MAPK pathways and inflammatory NF-ĸB pathway, and promotes increased shedding of EVs, antiangionenic factors, and inflammatory cytokines [41, 42].

Glutaminase is one of the key enzymes in cell metabolism. We have previously studied the effects of neuron, macrophage, and microglia GLS in brain injury, infection, and inflammation [5, 11, 24, 25, 29, 43]. Recently, we found that HIV-1 infection and immune activation increase EV release from macrophages and microglia [24]. Interestingly, GAC, an isoform of GLS, is released into the extracellular fluid primarily via EVs and it then induces neurotoxicity. Others found that larger vesicles (microvesicles) are sensitive to glutamine inhibition, as the introduction of a glutaminase inhibitor significantly disrupted their production in cancer cells [12]. However, due to the limitation of DLS technology, it could not analyze the number, concentration, and particle sizes of EVs. The work described here showed the EV releasing role of TNF-α by a mouse astrocyte model. The concentrations and diameters of EVs were detected by NTA, which provides accurate measurements. Our current studies found that TNF-α increased EV release in mouse astrocytes. Furthermore, the analysis of glutaminase isoforms revealed that TNF-α upregulated GAC expression in mouse astrocytes. Inhibition of GLS and ROS both could decrease the EV release which was mediated by TNF-α. We also investigated the level of EVs in Gls−/− mouse astrocytes. The concentration of EVs in Gls−/− astrocytes was ten-fold lower than wild type astrocytes, and the level of EVs in Gls−/− astrocytes did not change after TNF-α treatment.

Astrocytes use gliotransmitters to modulate neuronal function and plasticity. Recently, others discovered a fundamentally different form of long-term potentiation (LTP) that is induced by glial cell activation and mediated by diffusible, extracellular messengers, including D-serine and tumor necrosisfactor (TNF), and were spread widely in nociceptive pathways [44]. Astrocyte-derived EVs are heterogeneous in their composition and have been ascribed as having beneficial and detrimental functions, and they promise to be an exciting area of exploration. However, changes in the contents of EVs and its effect on other cells in the nervous system remain to be studied. Astrocyte-derived EVs have been implicated in the propagation of pathogenic proteins in neurodegenerative disorders [45], and they also can mediate neuroprotection [46]. In cancer, people have found that astrocyte-derived EVs induce PTEN suppression to foster brain metastasis, these findings highlighted an important plastic and tissue-dependent nature of metastatic tumor cells and a bi-directional co-evolutionary view of “seed and soil” hypothesis [47]. The cargo and the secretion mechanism of astrocyte-derived EVs may open the doors to a better understanding of how astrocytes impact on neuronal functions, and it may also provide us with new tools to compensate for cellular malfunctions under pathological conditions. Addressing this question poses one important challenge, which requires the development of techniques that induce EV secretion in vivo. Another major challenge to us is uncovering the relationship between glutaminase and EV release, but specifically packaged cargo and physiological function of EVs in vivo.

In summary, we have identified glutaminase as an important player in mediating EV over-release during inflammatory cytokines stimulation. EVs in inflammatory stress may be associated with the occurrence and development of the disease and cellular immune regulation. Uncovering the important role of glutaminase in inflammation-induced EV release, GLS may provide a potentially pathological mechanism in neuroinflammation, and a possible therapeutic target of inflammatory brain diseases.

## Conclusions

In summary, our studies further explored the question about the cellular mechanisms of EV release, implicating an important role of glutaminase. These new findings support the notable role of glutaminase and suggest a potential regulatory mechanism in which glutaminase may represent a promising therapeutic target that mediates CNS inflammation.

## Additional files

Additional file 1: Figure S1. Dynamic light scattering measurements demonstrate that treatment of mouse astrocytes with TNF-α does not affect the distribution of EVs. a Immunofluorescent staining for GFAP (red) in primary mouse astrocytes. Scale bars all indicated 50 μm. b A viability assay for astrocytes treated with TNF-α after 24 h. c Western Blot for calreticulin in astrocytes and EVs, calreticulin was a marker of endoplasmic reticulum, actin was a loading control. d EVs were isolated from serum-free culture of control and TNF-α-treated group after 24 h, and the size of EVs were determined by dynamic light scattering (DLS). The results were shown by intensity percent. (PDF 1048 kb)

Additional file 2: Figure S2. TNF-α induces GLS release from the mitochondria in mouse astrocytes. a After 72 h stimulation, mitochondria from mouse astrocytes were isolated, and then subjected to Western blotting analysis using anti-glutaminase and anti-ATP5A antibodies. ATP5A was a mitochondria marker and actin was a loading control. Gls−/− mouse astrocytes were treated with TNF-α for 48 h and then stained with DCFH-DA. Cells were washed with serum-free culture medium, and then subjected to fluorescence microscope (b) and fluorescent microplate reader (c). (PDF 769 kb)

## Abbreviations

AD: Alzheimer’s disease
Alix: ALG-2 interacting protein
ALS: Amyotrophic Lateral Sclerosis
BPTES: Bis-2-(5-phenylacetamido-1,3,4-thiadiazol-2-yl)ethyl sulfide
CNS: Central nervous system
CSF: Cerebrospinal fluid
DCFH-DA: 2,7-Dichlorodi-hydrofluoresceindiacetate
DLS: Dynamic light scattering
EVs: Extracellular vesicles
GAC: Glutaminase C
GLS: Glutaminase
GW4869: *N*,*N*’-Bis[4-(4,5-dihydro-1H-imidazol-2-yl)phenyl]-3,3’-p-phenylene-bis-acrylamide dihydrochloride
HPLC: High performance liquid chromatography
KGA: Kidney type glutaminase
NAC: *N*-Acetyl-l-cysteine
NTA: Nanoparticle tracking analysis
PD: Parkinson’s disease
PPMS: Primary progressive multiple sclerosis
ROS: Reactive oxygen species
SEM: Scanning electron microscopy
TEM: Transmission electron microscopy
TNF-α: Tumor necrosis factor-α
