# Supplementary figures and images for: TNF-α promotes extracellular vesicle release in mouse astrocytes through glutaminase
Source: J Neuroinflammation. 2017 Apr 20;14:87. doi: 10.1186/s12974-017-0853-2 (PMC5399318; doi:10.1186/s12974-017-0853-2)

A

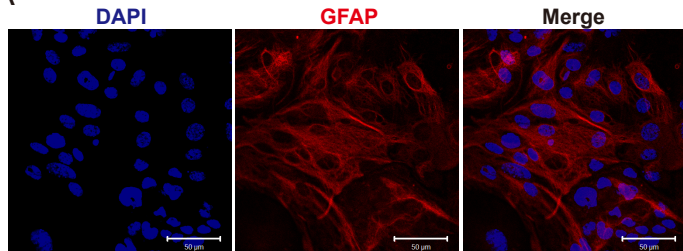

B

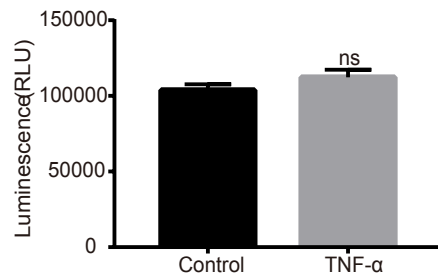

C

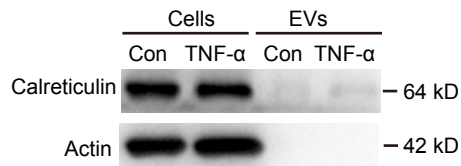

D

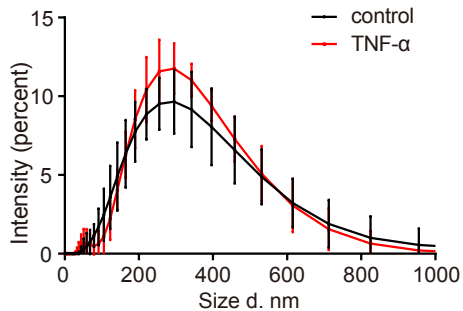

Supplement: Supplementary file 1 — Dynamic light scattering measurements demonstrate that treatment of mouse astrocytes with TNF-α does not affect the distribution of EVs. a Immunofluorescent staining for GFAP (red) in primary mouse astrocytes. Scale bars all indicated 50 μm. b A viability assay for astrocytes treated with TNF-α after 24 h. c Western Blot for calreticulin in astrocytes and EVs, calreticulin was a marker of endoplasmic reticulum, actin was a loading control. d EVs were isolated from serum-free culture of control and TNF-α-treated group after 24 h, and the size of EVs were determined by dynamic light scattering (DLS). The results were shown by intensity percent. (PDF 1048 kb) [file 12974_2017_853_MOESM1_ESM.pdf]

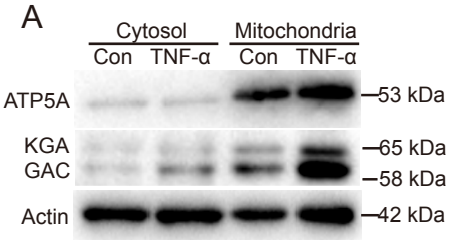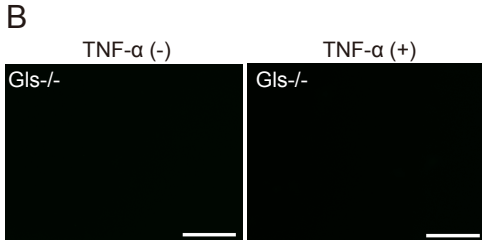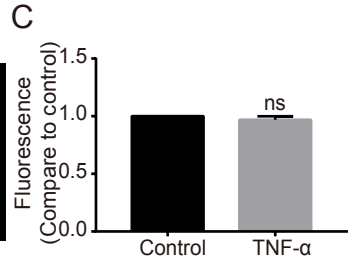

Supplement: Supplementary file 2 — TNF-α induces GLS release from the mitochondria in mouse astrocytes. a After 72 h stimulation, mitochondria from mouse astrocytes were isolated, and then subjected to Western blotting analysis using anti-glutaminase and anti-ATP5A antibodies. ATP5A was a mitochondria marker and actin was a loading control. Gls−/− mouse astrocytes were treated with TNF-α for 48 h and then stained with DCFH-DA. Cells were washed with serum-free culture medium, and then subjected to fluorescence microscope (b) and fluorescent microplate reader (c). (PDF 769 kb) [file 12974_2017_853_MOESM2_ESM.pdf]
